# Research Objectives, Statistical Analyses and Interpretation of Health-Related Quality of Life Data in Glioma Research: A Systematic Review

**DOI:** 10.3390/cancers12123502

**Published:** 2020-11-24

**Authors:** Marijke B. Coomans, Marthe C.M. Peeters, Johan A.F. Koekkoek, Jan W. Schoones, Jaap Reijneveld, Martin J.B. Taphoorn, Linda Dirven

**Affiliations:** 1 Department of Neurology, Leiden University Medical Center, 2333 ZA Leiden, The Netherlands; M.C.M.Peeters@lumc.nl (M.C.M.P.); J.A.F.Koekkoek@lumc.nl (J.A.F.K.); m.j.b.taphoorn@lumc.nl (M.J.B.T.); L.Dirven@lumc.nl (L.D.); 2 Department of Neurology, Haaglanden Medical Center, 2262 BA The Hague, The Netherlands; 3 Walaeus Library, Leiden University Medical Center, 2333 ZA Leiden, The Netherlands; J.W.Schoones@lumc.nl; 4 Department of Neurology and Brain Tumour Center Amsterdam, Amsterdam University Medical Center, 1007 MB Amsterdam, The Netherlands; jc.reijneveld@amsterdamumc.nl; 5 Stichting Epilepsie Instellingen Nederland (SEIN), 2103 SW Heemstede, The Netherlands

**Keywords:** brain tumor, quality of life, patient-reported outcome, questionnaire, HRQoL, PRO

## Abstract

**Simple Summary:**

Health-related quality of life (HRQoL) is an important outcome in glioma patients, as it reflects the patient’s perspective on their functioning and wellbeing through the disease course. The aim of our systematic review was to provide an overview of how HRQoL data is currently analyzed and interpreted in glioma studies. We found that the number of studies including HRQoL data increased in the past decade, but that assessment and analytical methods were highly variable. Ways to maximize information obtained with HRQoL questionnaires include appropriate and complementary analyses at both the group and individual level, comprehensive reporting of HRQoL results, and adherence to existing guidelines on the assessment, analysis, reporting and interpretation of patient-reported outcomes. This may ultimately result in high quality information that is relevant to inform physicians, patients and their relatives about the impact of the disease and its treatment on the patients’ functioning and well-being.

**Abstract:**

Background: Health-related quality of life (HRQoL) has become an increasingly important patient-reported outcome in glioma studies. Ideally, collected HRQoL data should be exploited to the full, with proper analytical methods. This systematic review aimed to provide an overview on how HRQoL data is currently evaluated in glioma studies, focusing on the research objectives and statistical analyses of HRQoL data. Methods: A systematic literature search in the databases PubMed, Embase, Web of Science and Cochrane was conducted up to 5 June 2020. Articles were selected based on predetermined inclusion criteria and information on study design, HRQoL instrument, HRQoL research objective and statistical methods were extracted. Results: A total of 170 articles describing 154 unique studies were eligible, in which 17 different HRQoL instruments were used. HRQoL was the primary outcome in 62% of the included articles, and 51% investigated ≥1 research question with respect to HRQoL, for which various analytical methods were used. In only 42% of the articles analyzing HRQoL results over time, the minimally clinical important difference was reported and interpreted. Eighty-six percent of articles reported HRQoL results at a group level only, and not at the individual patient level. Conclusion: Currently, the assessment and analysis of HRQoL outcomes in glioma studies is highly variable. Opportunities to maximize information obtained with HRQoL data include appropriate and complementary analyses at both the group and individual level, comprehensive reporting of HRQoL results in separate articles or supplementary material, and adherence to existing guidelines about the assessment, analysis and reporting of patient-reported outcomes.

## 1. Introduction

To evaluate the impact of a treatment strategy on outcomes in glioma patients, the most common malignant primary brain tumor [1], several outcome measures are needed. Besides traditional outcomes such as overall survival, progression-free survival and radiographic response, clinical outcome assessments (COAs) have become important in the evaluation of the full impact of a treatment strategy [2]. COAs reflect how patients feel, function or survive, and include among others patient-reported outcomes (PROs). Especially in glioma patients, in which the quality of life is at least as important as the duration of survival, PROs are particularly important as they provide the patients view on the disease burden and treatment effects [3]. An important PRO is health-related quality of life (HRQoL), a multidimensional concept comprising domains related to physical, mental, emotional and social functioning. Currently, many clinical trials include HRQoL outcomes alongside survival outcomes to assess the net clinical benefit of a treatment strategy, in which the benefits of the new treatment strategy in terms of prolonged (progression-free) survival are weighed against the possible side-effects and subsequent deterioration in the patient’s functioning and well-being measured with PROs or other COAs. Including different outcomes is necessary in glioma patients, because they experience a variety of symptoms and impaired functioning due to the treatment and its disease, including general cancer symptoms such as fatigue and mood disorders, but also central nervous system-specific symptoms such as seizures, focal neurological deficits, signs of elevated intracranial pressure including headache and neurocognitive deficits, which need to be assessed to provide a complete picture of the patient [4].

The increased use of HRQoL measures in clinical trials and other study designs, and the lack of (a proper use of) recent guidelines on the analysis, interpretation and reporting of PRO data has led to difficulties in drawing conclusions on the patient experience of a new cancer treatment, and hampers comparison of HRQoL results across similar trials [5,6]. Processing of HRQoL data is complex, because the instruments are typically multidimensional, including multiple symptoms and domains of functioning and wellbeing. In addition, different study designs and different choices in a priori planning of statistical analyses contribute to the variety of information that can be obtained with HRQoL assessments. First, the research objective determines the type of analysis; e.g., HRQoL data can be used as a prognostic indicator for survival, to determine the impact of a treatment on the patients’ functioning and well-being by comparing HRQoL scores between treatment arms, or to determine the association between HRQoL and other factors (e.g., the use of antiepileptic drugs). Moreover, HRQoL data can be analyzed at one specific point in time (i.e., cross-sectional) or over time (i.e., longitudinal). Lastly, HRQoL scores can be analyzed at the group level or at the individual patient level. For each research objective, different analytical methods could be used, which may result in different types of HRQoL information that is obtained, and may subsequently hamper comparison between studies that are similar in design and/or research question. We hypothesized that there might be a large variation in research objectives, statistical analyses and interpretation of HRQoL data, hampering comparison across studies.

The aim of this systematic review was to provide an overview on how HRQoL data is currently evaluated in glioma studies, focusing on the research objectives, statistical analyses and interpretation of HRQoL data. Better insights in current practices may inform the development or revision of guidelines to standardize the assessment, analysis and interpretation of HRQoL data in glioma studies, ultimately improving the value of HRQoL data in the evaluation of specific treatment strategies and facilitate comparability across studies.

## 2. Methods

### 2.1. Search Strategy

We used the methodology for screening and reporting as described in the Preferred Reporting Items for Systematic Reviews and Meta-Analyses (PRISMA) guidelines [7]. A literature search was performed in the electronical databases PubMed/Medline, Embase, Emcare, Cochrane, Web of Science, Academic Search premier and PsychINFO and included all publications from August 1999 up to 5 June 2020, as this was deemed a relevant time frame that would be representative on how HRQoL is currently evaluated in glioma studies. Our search strategy included search strings related to ‘brain tumor’ and ‘quality of life’ (Table S1 for the complete search strategy in PubMed).

### 2.2. Selection Criteria

Included articles were original peer-reviewed articles reporting HRQoL results in adult (≥18 years) glioma patients. As different definitions of HRQoL exist, the included articles had to meet the following aspects: HRQoL was measured as a multidimensional concept, covering both symptoms and functioning, and had to be measured from a patients’ perspective (i.e., a PRO measure). All types of studies were considered. Exclusion criteria were: articles not in English, studies with children only (<18 years), studies including both adults and children but without a subanalysis in adults, case studies, case series (*n* ≤ 10) as proper statistical analyses are not possible in such small populations. We also excluded (systematic) reviews that did not include individual patient data, study protocols, letters to the editor that did not include original results, comments/notes and conference abstracts. Studies reporting quality adjusted life years (QALYs) were only eligible if the use of a patient-reported outcome was explicitly mentioned. Two researchers (M.B.C. and M.C.M.P.) independently screened all titles/abstracts for eligibility. Possible eligible articles were screened full-text using the same predefined inclusion and exclusion criteria. In case of disagreement, a third reviewer (L.D.) was consulted. Included articles may include results from the same study population. Therefore, a clear distinction was made between studies and articles (i.e., publication from a certain study), to prevent certain outcomes from being reported twice (e.g., type of HRQoL instruments used).

### 2.3. Data Extraction

A study-specific data extraction form was developed and the following data was extracted per included article: year of publication, study design, number of included patients, number of females, median/mean age of the study population, HRQoL instrument used, research objective concerning HRQoL, statistical technique used to analyze the HRQoL data and a statement and interpretation of the minimally clinical important difference (MCID). HRQoL research objectives were classified as: comparison of mean scores between groups at one time point, association of HRQoL with another outcome, prediction/prognostic model (HRQoL as the outcome or as the predictor), comparison of HRQoL in one group over time (cross-sectional at each time point or longitudinal), and lastly comparison of mean scores between groups at multiple timepoints (again cross-sectional at each time point or longitudinal). In addition, we evaluated whether HRQoL was analyzed at the group and/or individual level (group level vs. individual level vs. both). For the classification into group/individual level we used the following criteria: for group level, mean (change) scores for HRQoL scales were calculated for all patients together. When analyzed at the individual patient level, either the proportion of patients that improved, deteriorated or remained stable on a specific scale had to be described, or the proportion of patients that deteriorated on all scales, improved or remained stable on all scales, or both deteriorated and improved.

## 3. Results

### 3.1. Search Results

**Figure 1:**
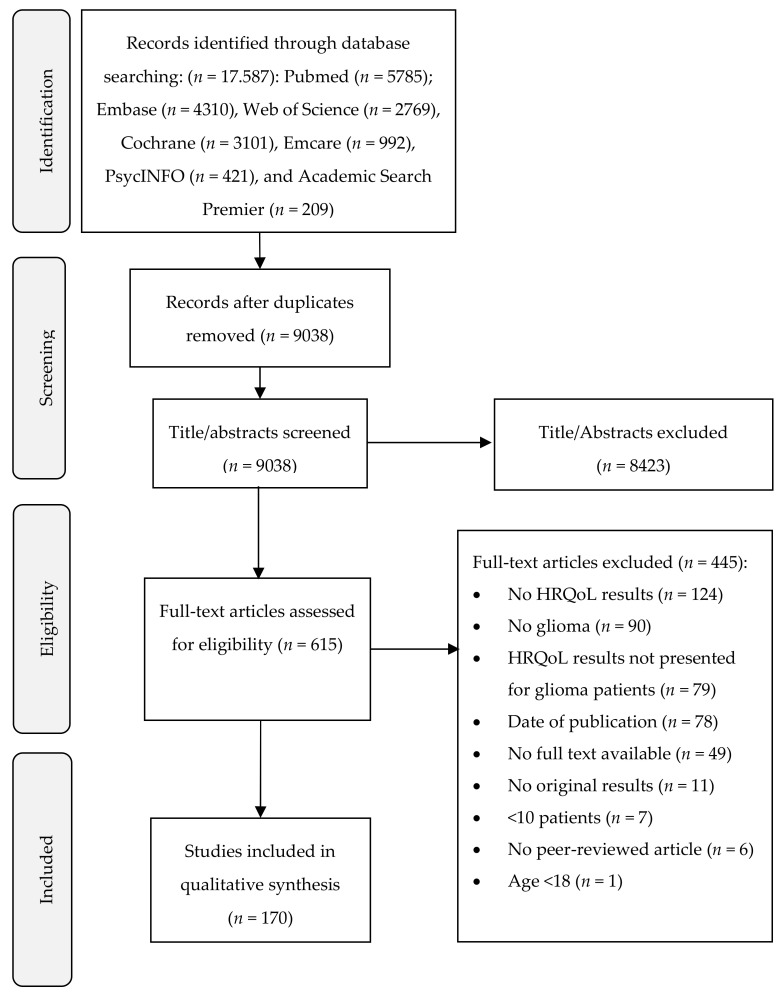
Flow diagram of study selection.

The literature search resulted in 9038 unique titles/abstracts, of which 170 were eligible according to our inclusion criteria (see Figure 1 for the article selection procedure and Table S2 for the complete reference list). The eligible articles comprised a large sample, with median of 80 patients per study (range: 10–5217). Most studies were observational cohort studies (35%), followed by interventional randomized and non-randomized studies (24% and 18%, respectively; Table 1). Most articles (51%) were published between 2015 and 2020, compared to 32% between 2009 and 2014, 9% between 2004 and 2009 and 8% between 1999 and 2003, reflecting an increase in the number of publications on this topic in the last years. HRQoL was the primary outcome in 105 articles (62%).

Importantly, there were multiple articles reporting on patients from one study. For example, EORTC trial 26951 resulted in four articles to be included in this review as they all investigated HRQoL-related questions [8,9,10,11]. Additionally, two meta-analyses with individual patient data, comprising results of 15 RCTs, were included [12,13]. In total, the 170 articles comprised 154 unique study populations, including 19.228 patients (range 10–845).

### 3.2. HRQoL Instruments

Most studies used a cancer-specific HRQoL instrument in combination with a brain tumor-specific instrument (70/154, 45%, Table 1). In total, 17 different instruments were identified in the eligible studies, of which the European Organization for Research and Treatment of Cancer (EORTC) quality of life questionnaire core-30 (QLQ-C30), the Brain Neoplasm module (QLQ-BN20) and the Functional Assessment of Cancer Therapy-brain (FACT-Br) were most often used (in 33%, 31% and 14% of the studies, respectively).

### 3.3. Research Objectives

About half of the included articles addressed more than one research objective with respect to HRQoL (51%), adding up to a total of 279, although not unique, research objectives (Table 1). A frequent research objective was to study HRQoL over time in one group (*n* = 55/170, 32%), or in 2 or more groups (*n* = 83/170, 49%). When analyzing HRQoL scores between groups over time, most articles (*n* = 49/83, 59%) used cross-sectional methods to compare mean scores at multiple time points, whereas the other 34 articles used longitudinal methods. Similarly, when analyzing mean scores over time in one group, both cross-sectional (i.e., calculated for each time point separately; *n* = 41/55) and longitudinal methods (i.e., including all time points in one analysis; *n* = 14/55) were used. Twelve out of 83 articles combined cross-sectional and longitudinal methods to compare groups over time. In 56/170 articles, HRQoL scores were analyzed at one time point, either compared between groups (*n* = 37/56) or compared with a norm group (*n* = 19/56).

The association of HRQoL with another outcome was another frequently used research objective (*n* = 66/170 articles, 39%). The association between HRQoL (*n* = 40/66) and patient, clinical or treatment-related variables was most commonly evaluated, i.e., sociodemographic and clinical variables such as age, tumor volume or extent of resection or performance status (clinician-reported outcome). In 20/66 articles, the association between HRQoL and other PROs were determined, for example mood, coping or fatigue. In the remaining 6/66 articles HRQoL was associated with performance outcomes (i.e., neurocognitive functioning), HRQoL of proxies or both clinical and PROs data. In 19/170 articles (11%), prediction models were constructed including HRQoL data. In 6 of these articles HRQoL was the dependent variable, while in the other articles HRQoL data was included as an independent (predictor) variable for example for survival or performance status.

### 3.4. Statistical Methods

Different statistical methods were used for each research objective (Table 2). When comparing HRQoL at one time point between groups (*n* = 56 articles), Mann–Whitney U/Wilcoxon signed rank/Kruskal–Wallis tests were most often used (*n* = 17/56, 30%), followed by a Student’s *t*-test (*n* = 12/56, 21%) descriptive analysis (*n* = 11/56, 20%) and ANOVA/ANCOVA (*n* = 7/56, 13%), depending on the distribution of the variables and the number of compared groups. When studying HRQoL over time with cross-sectional research objectives, i.e., comparing mean scores at multiple time-points in one or more groups (*n* = 90 articles), Mann–Whitney U/Wilcoxon signed rank/Kruskal–Wallis tests and descriptive analyses (both *n* = 25/90, 28%) and Student’s *t*-test (*n* = 19/90, 21%) were again most often used. In the articles with longitudinal research objectives, evaluating HRQoL scores over time either between groups or in one group (*n* = 48 articles), mixed effect models were most often used (*n* = 20/48, 42%), followed by survival analysis (*n* = 10/48, 21%; for example calculating the time to HRQoL deterioration and deterioration-free-survival time). Joint models, reliable change models and regression analyses calculating change scores over time are other examples of longitudinal methods used (Table 2).

### 3.5. Group and Individual Level

Most articles reported HRQoL results at the group level only (146/170, 86%), whereas only a few reported results at the individual patient level only (6/170, 4%) or at both group and individual level (18/170, 10%). In the articles reporting results at the individual level, mostly the percentage of patients that improved or decreased on a specific scale (‘individual scale level’) were calculated, whereas only one article reported the results at the individual patient level including all scales simultaneously. Of note, the six articles reporting results only at the individual level (both patient and scale level) were particularly small phase I/II trials, including only 15–29 patients [14,15,16,17,18,19,20]. Thirteen of the 18 articles that reported HRQoL both at the group and individual scale level were interventional (non-)randomized studies including 65–902 patients.

### 3.6. Minimally Clinical Important Difference

The MCID was both described and interpreted in 58 (42%) of the 138 longitudinally analyzed studies comparing HRQoL results over time in one or more groups. In the remaining articles, either the MCID was not described at all, even though they are available for most instruments, or results were not interpreted according the MCID. This means that only the statistical significance of results was reported, and not if these differences were also clinically relevant, which would provide more valuable results.

## 4. Discussion

In this systematic review we provided an overview on how HRQoL data is currently measured, analyzed and reported in glioma studies. Our review showed an increase in studies using HRQoL instruments in the past decade, particularly in the past five years. The 170 identified articles resulted from 154 different studies in glioma patients, where half of the articles (83/170, 49%) focused on one research objective with respect to HRQoL. The limited word count of articles describing all trial outcomes may have hampered a proper description and reporting of the specific HRQoL hypothesis and analyses, and also raises the question whether all available data with regard to HRQoL have currently been analyzed optimally, and if additional questions might have been answered if all available HRQoL data had been used to the fully. To obtain all relevant information with respect to HRQoL data, separate reporting of HRQoL results could be a solution. Previous reviews on the level of reporting of HRQoL findings in oncological trials reported that a description of HRQoL results in a separate article was associated with better reporting and higher quality description [21,22].

Furthermore, even if someone is interested in answering one specific research question, e.g., HRQoL over time, it may be useful to combine multiple analytical methods as these may provide different types of complementary information. For example, when comparing mean HRQoL scores between groups over time, both longitudinal and cross-sectional methods may be useful, which was only done in 12/170 (7%) of the included articles. Moreover, whatever the research question is, it is important that appropriate statistical methods are used. Particularly those statistical techniques that take into account drop-out over time are sometimes overlooked while they are very important, since missing data is an inherent problem in glioma trials and is often not completely at random but dependent on the patient’s clinical status (e.g., patients drop out because of poor health or disease progression [6]).

Further, we found that the minimally clinical important difference in HRQoL results, representing the smallest changes/differences in HRQoL scores that are perceived to be clinically relevant, was only applied in 42% of the articles evaluating HRQoL over time. When interpreting within-group or between-group change scores in HRQoL, not only statistically significant differences should be considered, but also the clinical relevance of these differences. Interpreting HRQoL scores via statistical significance only may be misleading because small differences in mean scores can be statistically significant, particularly in large samples, even when clinical relevance is absent. Although MCIDs exist for several questionnaires, some are too general and may therefore be less appropriate. For example, in general the MCID used for scales of EORTC measures is ≥10 points, but may be too simplistic, as it does not differentiate between the different scales, the direction of change (improvement versus deterioration) and is not applicable to all cancer populations [23,24]. Therefore, for the EORTC QLQ-C30, MCIDs for each scale are currently being developed for different cancer sites [25].

The results further showed that only 24 articles reported HRQoL results at the individual patient level. Such analyses may be of high clinical relevance as they could result in a different conclusion about the impact of treatment when compared to analysis at the group level. One study indeed showed that analysis at the group level resulted in the interpretation that HRQoL scores were stable over time, while analysis at the individual patient resulted in a different conclusion, i.e., that a large proportion of the patients deteriorated on certain HRQoL scales, and improved on other scales [8]. The number of patients deteriorating and improving on a certain scale cancelled out, suggesting that HRQoL remained stable, while in fact patients experienced large changes in their perceived HRQoL. In addition to analyzing change in HRQoL at the individual scale level, analyzing HRQoL at the individual patients levels considering all scales simultaneously, for example with a heatmap [26], may provide further clinically relevant information. This additional information may better inform patients and physicians on the impact of treatment on their functioning and wellbeing.

Guidelines that address the use of proper statistical methods and clear description of the results exist, and may help to exploit HRQoL data to full and make HRQoL results easily interpretable. In the included RCTs in our study that have been published in the last five years, only 14% (2/14) reported making use of the CONSORT-PRO guideline and another 36% (5/14) used the CONSORT diagram without further statement. No other guidelines were described. Although sparsely described, several guidelines have already been published that address the collection, analysis, interpretation and reporting of PRO results. For interventional trials, several guidelines and initiatives are available or ongoing. The SPIRIT-PRO guidelines are available to guide researchers when developing a trial protocol including PROs as a primary or secondary endpoint, and addresses important issues such as study objectives and selection of HRQoL measures [27,28]. The SISAQOL initiative provides guidance on the analysis and interpretation of PROs in cancer clinical trials [29]. With regard to the reporting of HRQoL data, the CONSORT-PRO guidelines, the only guideline that was described in our identified RCTs in the last five years, postulates guidance in reporting PROs in clinical trials [30]. Considering the use of instruments to measure HRQoL in neuro-oncological studies, the RANO-PRO initiative was initiated, aiming to provide guidance on the use of specific PROs specific in neuro-oncological patient populations [31]. In a broader sense, the PROTEUS project intends to promote the implementation and dissemination of methodological tools that have been developed to optimize the assessment and reporting of PROs in clinical trials [32]. For observational studies on the other hand, less guidelines are available. Nevertheless, the STRATOS initiative aims to provide guidance in the design and analysis of observational studies, reacting on frequent analytical errors and the rapid development of methodological methods [33]. Using these guidelines may result in high quality collection, measurement, analyses, interpretation and reporting of HRQoL data. Moreover, meta-analyses that involve individual patient data (IPD analyses) of multiple studies may provide additional clinically relevant information and a greater depth of understanding HRQoL data than is possible from single studies [34]. Therefore, IPD analyses represent a unique resource for secondary hypothesis testing and exploratory analyses, which could provide opportunities to answer relevant questions with respect to HRQoL in glioma patients in the future.

## 5. Conclusions

In conclusion, although the number of glioma articles that included HRQoL as the endpoint has increased over the years, the analytical methods and reporting of HRQoL results in observational studies and RCTs is highly variable. Opportunities to maximize information obtained with HRQoL data include appropriate and complementary analyses at both the group and individual level, comprehensive reporting of HRQoL results in separate articles or supplementary material and adherence to existing guidelines on the assessment, analysis, reporting and interpretation of PROs. Ultimately, this may result in more complete information that is relevant to inform patients about the impact of a treatment on their functioning and well-being.

## Figures and Tables

**Table 1:** Characteristics of the included articles and studies.

**Characteristics of Included Articles (*n* = 170)**	**Articles (*n*, %)**
Published in 2015–2020 Published in 2009–2014 Published in 2004–2009 Published in 1999–2003	87 (51) 54 (32) 15 (9) 14 (8)
Study design	
Cross-sectional (i.e., one timepoint only) Observational cohort Observational case-control Interventional randomized Interventional non-randomized	27 (16) 60 (35) 11 (7) 41 (24) 31 (18)
Research objectives concerning HRQoL (≥1 objective per article possible, *n* = 279)	
Compare mean scores between groups at one time point Association with other outcome (i.e., fatigue, mood) Prediction model: HRQoL as covariate Prediction model: HRQoL as outcome Compare over time in one group: cross-sectional Compare over time in one group: longitudinal Compare mean scores between groups at multiple time points: cross-sectional Compare mean scores between groups at multiple time points: longitudinal	56 (33) 66 (39) 13 (8) 6 (4) 41 (24) 14 (8) 49 (29) 34 (20)
1 research objective regarding HRQoL ≥2 research objectives regarding HRQoL	83 (49) 87 (51)
MCID mentioned and interpreted in longitudinal studies (*n* = 138) that compared HRQoL over time in one or more groups	58 (42)
Analyses at group level only Analyses at both group and individual level Analyses at individual level only	146 (86) 18 (10) 6 (4)
**Characteristics of Included Studies (*n* = 154)**	**Studies (*n*, %)**
HRQoL instrument of included studies	
Generic only Cancer-specific only Brain tumor-specific only Generic and brain tumor-specific Cancer-specific and brain tumor-specific	26 (17) 15 (10) 37 (24) 6 (4) 70 (45)
HRQoL instrument used (≥1 instrument per article possible)	
EORTC QLQ-C30	87 (33)
EORTC QLQ-BN20 FACT-Br SF-36	81 (31) 37 (14) 13 (5)
FACT-G	9 (3)
EQ-5D MDASI Other	12 (5) 10 (4) 13 (5)

RCT: Randomized controlled trial, HRQoL: Health-related quality of life, EORTC QLQ-C30: European Organization for Research and Treatment of Cancer Quality of life Questionnaire 30, EORTC QLQ-BN20: European Organization for Research and Treatment of Cancer Quality of life Questionnaire Brain Neoplasm 20, FACT-G: Functional Assessment of Cancer Therapy General, FACT-Br: Functional Assessment of Cancer Therapy Brain, SF-36: Medical outcomes study 36 Short Form health survey, EQ-5D: EuroQol 5D, MDASI: MD Anderson Symptom Inventory.BN20: European Organization for Research and Treatment of Cancer Quality of life Questionnaire Brain Neoplasm 20, FACT-G: Functional Assessment of Cancer Therapy General, FACT-Br: Functional Assessment of Cancer Therapy Brain, SF-36: Medical outcomes study 36 Short Form health survey, EQ-5D: EuroQol 5D, MDASI: MD Anderson Symptom Inventory.

**Table 2:** Statistical tests per HRQoL research objective.

Research Objectives Concerning HRQoL	*n*	Statistical Test (≥1 Test Per Research Objective Possible)
*One timepoint*		
Association of HRQoL with other outcome (i.e., fatigue, mood)	66	Spearman/Pearson correlation analyses (*n* = 27) Regression analysis (*n* = 21) Linear model (*n* = 6) Mann–Whitney U/Kruskal–Wallis/Wilcoxon test (*n* = 4) ANOVA/ANCOVA (*n* = 2) Unknown (*n* = 2) Actor-partner independence model (*n* = 1) Chi square test (*n* = 1) Bland–Altman (*n* = 1) Student’s *t*-test (*n* = 1) Rank-sum test (*n* = 1) Bootstrap mediation analyses (*n* = 1) Descriptive (*n* = 1)
Compare mean scores between groups at one time point	56	Mann–Whitney U test/Wilcoxon rank sum test/Kruskal–Wallis test (*n* = 17) Student’s *t*-test (n = 12) Descriptive (*n* = 11) ANOVA/ANCOVA (*n* = 7) Chi-square test (*n* = 3) Unknown (*n* = 2) Paired signed-rank test (*n* = 1) Kolmogorov–Smirnov test (*n* = 1)
Prediction model: HRQoL as covariate	13	Cox model (*n* = 11) Regression analysis (*n* = 2)
Prediction model: HRQoL as outcome	9	Regression analysis (*n* = 5) Cox model (*n* = 2) Principal components analysis (*n* = 1) Unknown (*n* = 1)
*Multiple timepoints: cross-sectional*		
Compare mean scores between groups	49	Descriptive (*n* = 16) Unknown (*n* = 11) Mann–Whitney U/Kruskal–Wallis/Wilcoxon test (*n* = 9) Student’s *t*-test (*n* = 7) Chi-square test (*n* = 3) ANOVA (*n* = 2) Reliable change index (*n* = 1) Area under the curve (*n* = 1) Fishers exact test (*n* = 1) Difference-in-difference approach (*n* = 1)
Compare HRQoL over time in one group	41	Mann–Whitney U/Kruskal–Wallis/Wilcoxon test (*n* = 16) Student’s *t*-test (*n* = 12) Descriptive (*n* = 9) Unknown (*n* = 3) Chi-square test (*n* = 1) ANOVA/ANCOVA (*n* = 1)
*Multiple time-points: longitudinal*		
Compare mean scores between groups	34	Mixed-effect models (*n* = 14) Survival analysis (*n* = 9) Other linear models (*n* = 5) Joint model (*n* = 2) Regression analysis (*n* = 2) Reliable change index (*n* = 1) GEE models (*n* = 1) Two-sample proportion test (*n* = 1) Descriptive (*n* = 1)
Compare HRQoL over time in one group	14	Descriptive (*n* = 7) Mixed effects model (*n* = 6) Regression model (*n* = 4) Area under the curve (*n* = 2) Reliable change index (*n* = 1) Survival analysis (*n* = 1)

In case the association between HRQoL and another outcome was determined (*n* = 66 articles), Pearson and Spearman correlational analyses were most often used (*n* = 27/66, 41%), depending on the distribution of the included variables, followed by linear and logistic regression analyses (*n* = 21/66, 32%). Finally, Cox proportional hazard models (*n* = 13, 59%), linear and logistic regression models (*n* = 7, 32%) and principal components analyses (*n* = 1, 5%) were used to construct prediction models. In 3/13 articles using Cox proportional hazard models, the added prognostic value of HRQoL compared to known clinical variables was calculated using the C-index. Of note, in 19/170 (11%) articles, the statistical methods were not described.

## Supplementary Materials

The following are available online at https://www.mdpi.com/2072-6694/12/12/3502/s1, Table S1: Search strategy, Table S2: Overview of 170 included articles.
