# Supplementary material for: Research Objectives, Statistical Analyses and Interpretation of Health-Related Quality of Life Data in Glioma Research: A Systematic Review
Source: Cancers (Basel). 2020 Nov 24;12(12):3502. doi: 10.3390/cancers12123502 (PMC7760401; doi:10.3390/cancers12123502)
Supplement: Supplementary file 1 [file cancers-12-03502-s001.pdf]

Table S1. Search strategy

(("Glioma"[Mesh] OR glioma*[tw] OR neuroglioma*[tw] OR xanthoastrocytoma*[tw] OR xantoastrocytoma*[tw] OR astrocytoma*[tw] OR astro-cytoma*[tw] OR astroglioma*[tw] OR astro-glioma*[tw] OR oligoastrocytoma*[tw] OR oligoastro-cytoma*[tw] OR glioblastom*[tw] OR glio-blastom*[tw] OR oligodendroglioma*[tw] OR oligo-dendroglioma*[tw] OR oligo-dendroglioma*[tw] OR oligodendroblastoma*[tw] OR oligo-dendro-blastoma*[tw] OR oligo-dendroblastoma*[tw] OR oligoden-droblastoma*[tw] OR oligodendro-blastoma*[tw] OR ependymom*[tw] OR subependymom*[tw] OR ganglioglioma*[tw] OR medulloblastoma*[tw] OR gliosarcoma*[tw] OR glio-sarcoma*[tw] OR glial tumour*[tw] OR glial tumor*[tw] OR glial neoplasm*[tw] OR "glial cancer"[tw] OR "glial carcinoma"[tw] OR "glial carcinomas"[tw] OR "glial malignancy"[tw] OR "glial malignancies"[tw] OR neuroglial tumour*[tw] OR neuroglial tumor*[tw] OR neuroglial neoplasm*[tw] OR "neuroglial cancer"[tw] OR "neuroglial cancers"[tw] OR "neuroglial carcinoma"[tw] OR "neuroglial carcinomas"[tw] OR "neuroglial malignancy"[tw] OR "neuroglial malignancies"[tw] OR "LGG"[tw] OR "HGG"[tw] OR "DLGG"[tw] OR "Brain Neoplasms"[Mesh] OR "brain malignancy"[tw] OR "brain malignancies"[tw] OR "malignant primary brain"[tw] OR "malignant brain"[tw] OR "glial tumour"[tw] OR "glial tumor"[tw] OR "glial neoplasm"[tw] OR "glial cancer"[tw] OR "glial carcinoma"[tw] OR "glial carcinomas"[tw] OR "glial malignan"[tw] OR "neuroglial tumour"[tw] OR "neuroglial tumor"[tw] OR "neuroglial neoplasm"[tw] OR "neuroglial cancer"[tw] OR "neuroglial cancers"[tw] OR "neuroglial carcinoma"[tw] OR "neuroglial carcinomas"[tw] OR "neuroglial malignancy"[tw] OR "neuroglial malignancies"[tw] OR "brain neoplasms"[tw] OR "brain tumour"[tw] OR "brain tumor"[tw] OR "cns tumour"[tw] OR "cns tumor"[tw] OR "central nervous system tumour"[tw] OR "central nervous system tumor"[tw] OR "intracranial tumour"[tw] OR "intracranial tumor"[tw] OR "cerebral tumour"[tw] OR "cerebral tumor"[tw] OR "intracerebral tumour"[tw] OR "intracerebral tumor"[tw] OR "glial tumour"[tw] OR "glial tumor"[tw] OR "neuroglia tumour"[tw] OR "neuroglia tumor"[tw] OR "tumour of brain"[tw] OR "tumor of brain"[tw] OR "tumours of brain"[tw] OR "tumors of brain"[tw] OR "tumour of the brain"[tw] OR "tumor of the brain"[tw] OR "tumours of the brain"[tw] OR "tumors of the brain"[tw] OR "brain tumour"[tw] OR "brain tumor"[tw] OR "Central Nervous System Neoplasms"[Mesh] OR "tumour of cns"[tw] OR "tumor of cns"[tw] OR "tumours of cns"[tw] OR "tumors of cns"[tw] OR "tumour of the cns"[tw] OR "tumor of the cns"[tw] OR "tumours of the cns"[tw] OR "tumors of the cns"[tw] OR "cns tumour"[tw] OR "cns tumor"[tw] OR "tumour of central nervous system"[tw] OR "tumor of central nervous system"[tw] OR "tumours of central nervous system"[tw] OR "tumors of central nervous system"[tw] OR

"tumour of the central nervous system"[tw] OR " tumor of the central nervous system"[tw] OR "tumours of the central nervous system"[tw] OR "tumors of the central nervous system"[tw] OR "central nervous system tumour"[tw] OR "central nervous system tumor"[tw] OR "intracranial tumour"[tw] OR "intracranial tumor"[tw] OR "cerebral tumour"[tw] OR "cerebral tumor"[tw] OR "intracerebral tumour"[tw] OR "intracerebral tumor"[tw] OR "cns neosplasm "[tw] OR "intracranial neoplasm"[tw] OR "cerebral neoplasm"[tw] OR "intracerebral neoplasm"[tw] OR "glial neoplasm"[tw] OR "neoplasm of brain"[tw] OR "neoplasms of brain"[tw] OR "neoplasm of the brain"[tw] OR "neoplasms of the brain"[tw] OR "brain neoplasm"[tw] OR "neoplasm of cns"[tw] OR "neoplasms of cns"[tw] OR "neoplasm of the cns"[tw] OR "neoplasms of the cns"[tw] OR "cns neoplasm"[tw] OR "neoplasm of central nervous system"[tw] OR "Neoplasms, Nerve Tissue"[Mesh]) AND ("Quality of Life"[mesh] OR "quality of life"[tw] OR "life quality"[tw] OR "HRQoL"[tw] OR "HRQL"[tw] OR "QoL"[tw] OR "Health Status"[Mesh:NoExp] OR "health status"[tw] OR "health level"[tw] OR "health levels"[tw] OR "health outcomes"[tw] OR "health outcome"[tw] OR "patient outcomes"[tw] OR "patient outcome"[tw] OR "Patient Outcome Assessment"[Mesh] OR "patient reported outcomes"[tw] OR "patient reported outcome"[tw] OR "PROs"[tw] OR "PROM"[tw] OR "PROMs"[tw]OR "symptom distress"[tw] OR "symptom burden"[tw] OR "Symptom Assessment"[Mesh] OR "symptom assessment"[tw] OR "functional status"[tw] OR "patient reported symptom"[tw] OR "patient reported symptoms"[tw])) AND english[la] NOT ("Animals"[mesh] NOT "Humans"[mesh]) NOT (("review"[ptyp] OR "review"[ti] OR systematic[sb] OR "case reports"[ptyp] OR "case report"[ti]) NOT ("Clinical Study"[ptyp] OR "trial"[ti] OR "RCT"[ti])) NOT (("Infant"[mesh] OR "Child"[mesh] OR "Adolescent"[mesh]) NOT "Adult"[mesh])

Table S2. Overview of 170 included articles

Included study	Study design	Year published	N	Age (mean/median)	N female	HRQoL primary or secondary endpoint	Research objective with regard to HRQoL	Statistical technique	HRQoL instrument	HRQoL measured at individual or group level	MCID described
Piil[1]	Cross-sectional	2019	13	56 (median)	7	Primary	Comparison with normgroup	Descriptive	FACT-G, FACT-Br	Group	No
Haraldseide[2]	Observational cohort	2019	114	64 (median)	36	Primary	Prognostic model (HRQoL as prognostic factor for survival)	Cox regression analyses	EQ-5D 3L, EORTC QLQ-C30 and EORTC QLQ-BN20	Group	No
Vaitkiene[3]	Observational cohort	2019	34	Unknown	23	Primary	Correlation between HRQoL and miR-34a expression	Spearman correlation analyses	EORTC QLQ-C30, EORTC QLQ-BN20	Group	No
Mukherjee[4]	Observational cohort	2019	145	49 (median)	62	Secondary	Comparison between resected and non-resected patients over time cross-sectionally	Chi-square test	SF-36	Group	Yes
Randazzo[5]	Observational cohort	2019	845	50 (median)	285	Primary	Association between the use of complementary treatment and HRQoL	(1) Kruskall-Wallis test<br>(2) Descriptive	FACT-Br	Group	No
Hansen[6]	Cross-sectional	2019	81	56 (mean)	27	Primary	Differences in HRQoL between patients with left and right tumor lateralization at one timepoint	Students T-test	EORTC QLQ-C30, EORTC QLQ-BN20	Group	No
Coomans[7]	Observational Cohort	2019	5217	53 (mean)	2005	Primary	(1) Comparison between patients with and without baseline HRQoL form<br>(2) Prognostic model, HRQoL as prognostic factor for survival	(1) Chi-square test, Students T-test<br>(2) Cox proportional hazard model, C-index	EORTC QLQ-C30, EORTC QLQ-BN20	Group	Yes

Onken[8]	Cross-sectional	2019	30	50 (mean)	10	Secondary	Comparison between patients and control group	(1) Students T-test	EORTC QLQ-C30, EORTC QLQ-BN20	Group	No
Renovanz[9]	Cross-sectional	2018	232	52 (mean)	103	Secondary	Association between HRQoL and patients' requested support	(1) Spearman correlation analyses<br>(2) multivariate logistic regression analyses	EORTC QLQ-C30	Group	No
Gabel[10]	Cross-sectional	2019	79	52 (mean)	50	Primary	(1) Prevalence of HRQoL deficits assessed with several questionnaires<br>(2) Difference in HRQoL between LGG and HGG	(1) Descriptive<br>(2) T-tests	PROMIS, NEURO-QOL	Group	No
Li[11]	Cross-sectional	2019	330	Unknown	142	Primary	(1) Association between post-traumatic stress symptoms and HRQoL<br>(2) Moderating effects of coping strategies on HRQoL	(1) Confirmatory factor analyses<br>(2) Pearson correlation analyses<br>(3) Hierarchical regression analyses	FACT-Br	Group	No
Dirven[12]	Interventional randomized	2020	28	37 (mean)	11	Primary	(1) Difference between two groups over time	(1) Descriptive<br>(2) Wilcoxon signed rank test	EORTC QLQ-C30, EORTC QLQ-BN20	Group and individual	Yes
Scartoni[13]	Observational cohort	2020	33	53 (median)	8	Primary	(1) Difference over time in one group<br>(2) Association with treatment characteristics over time	(1) Linear mixed models<br>(2) Multivariate linear mixed models	EORTC QLQ-C30, EORTC QLQ-BN20	Group	Yes
Faarup[14]	Observational case-control	2018	40	66 (mean)	10	Primary	Comparison with control group over time	Difference-in-difference approach	WHOQOL-BREF	Group	No

Wick[15]	Interventional cohort	2019	84	58 (median)	33	Primary	Comparison between groups over time	Survival analyses; time-to-deterioration	EORTC QLQ-C15 PAL, QLQ-BN20	Group	Yes
Dirven[16]	Interventional cohort	2019	195	Unknown	85	Primary	(1) Comparison between groups over time<br>(2) Association with radiation volumes<br>(3) Change from baseline	(1) Descriptive<br>(2) Logistic regression analyses<br>(3) Linear mixed models	EORTC QLQ-C30, EORTC QLQ-BN20	Group and individual	Yes
Bo [17]	Observational case-control	2020	47	43 (mean)	18	Secondary	Change in HRQoL in one groups at two timepoints	Descriptive	EQ-5D	Group and individual	Yes
Jacobs[18]	Observational case-control	2019	181	53 (mean)	97	Primary	(1) Comparison with normgroup<br>(2) Correlation with other outcomes<br>(3) Prognostic model, HRQoL as outcome	(1) Descriptive<br>(2) Pearson correlation<br>(3) Multivariable linear regression analyses	FACT-G	Group and individual	Yes
Kohler[19]	Cross-sectional	2019	74	54 (mean)	43	Primary	(1) Association with social support<br>(2) Assess whether the association is mediated by patients' disclosure behavior	(1) Pearson correlation analyses<br>(2) Linear regression analyses<br>(3) Bootstrap mediation analyses	SF-8	Group	No
Umezaki[20]	Cross-sectional	2020	76	51 (median)	35	Primary	(1) Comparison with normgroup<br>(2) Comparison of subgroups<br>(3) Association between symptoms	(1) Students T-test<br>(2) Chi-square test<br>(3) Uni and multivariable regression analyses	EORTC QLQ-C30, EORTC QLQ-BN20	Group	Yes
Weller[21]	Interventional randomized	2019	141	58 (mean)	Unkown	Primary	(1) Difference between groups over time	(1) Linear mixed models<br>(2) Multivariable cox analyses	EORTC QLQ-C30, EORTC QLQ-BN20	Group	Yes

							(2) Time to first deterioration				
Coomans[22]	Observational cohort	2019	4307	54 (mean)	2096	Primary	(1) Compare baseline HRQoL scores with normgroup<br>(2) Association with symptoms<br>(3) Association of symptoms with functioning scales	(1) Descriptive<br>(2) Spearman correlation analyses<br>(3) Partial network correlation analyses<br>(4) Hierarchical cluster analyses<br>(5) Multivariable linear regression analyses	EORTC QLQ-C30, EORTC QLQ-BN20	Group	Yes
Sagberg[23]	Observational cohort	2019	170	61 (mean)	62	Primary	(1) Compare differences between groups<br>(2) Compare HRQoL across tumor locations	(1) Mann Whitney U, T-tests<br>(2) Voxel based lesion mapping	EQ-5D	Group	Yes
Khatri[24]	Observational cohort	2019	103	43 (mean)	Unknown	Primary	(1) Compare baseline HRQoL scores between groups<br>(2) Compare HRQoL scores between groups over time	(1) Descriptive<br>(2) Students T-test	EQ-5D, SF-36	Group	Yes
Kumar[25]	Interventional cohort	2019	50	50 (mean)	19	Secondary	(1) Differences in HRQoL over time between groups	(1) General linear model	EORTC QLQ-C30, EORTC QLQ-BN20	Group	Yes
Kim[26]	Interventional cohort	2020	223	54 (median)	119	Primary	Differences in HRQoL over time between groups	Unknown	EQ-5D, FACT-Br	Group	Yes
Dutz[27]	Interventional cohort	2019	62	Unknown	23	Primary	(1) Differences between baseline ans follow-up scores (cross-sectional)<br>(2) Differences between baseline and follow-up (longitudinally)	(1) Wilcoxon<br>(2) Mixed effect models<br>(3) Spearman correlations	EORTC QLQ-C30, EORTC QLQ-BN20	Group	Yes

							(3) Correlation between outcomes				
Renovanz[28]	Observational cohort	2020	309	55 (mean)	130	Primary	(1) Compare HRQoL scores between two groups<br>(2) Association with clinical variables	(1) Descriptive<br>(2) Multivariable regression analyses	EORTC QLQ-C30, EORTC QLQ-BN20	Group	Yes
Jiang[29]	Observational cohort	2019	219	42 (mean)	83	Secondary	(1) Compare HRQoL scores between two groups at different time points<br>(2) Prediction model: HRQoL as covariate for outcome PTSS	(1) MANOVA<br>(2) Multivariate Cox regression model	SF-36	Group	No
Acquaye[30]	Cross-sectional	2019	23	53 (median)	12	Primary	(1) Comparison of interviews and MDASI-BT	(1) Descriptive	MDASI-BR	Group and individual	No
Milbury[31]	Interventional randomized	2020	20	Unknown	10	Secondary	(1) Comparison of change scores over time between groups	(1) General linear model	SF-36	Group	Yes
Hansen[32]	Interventional randomized	2020	64	54 (mean)	20	Primary	(1) comparison of HRQoL over time between groups	(1) Multiple regression model	EORTC QLQ-C30, EORTC QLQ-BN20	Group	Yes
Tabrizi[33]	Interventional cohort	2019	20	38 (median)	7	Secondary	(1) change in HRQoL over time in one group	(1) Linear mixed models	FACT-G, FACT-Br	Group	No
Affronti[34]	Observational cohort	2019	15	Unknown	11	Primary	Change in HRQoL over time in one group	Descriptive	FACT-G, FACT-Br	Group	No
Sacher[35]	Observational case-control	2018	45	53 (mean)	16	Primary	Interaction between HRQoL, mood and depression	Multiple linear regression analyses	SF-12	Group	No
Baumstark[36]	Observational cohort	2018	38	64 (median)	14	Primary	(1) Comparison between scores of caregivers and patients<br>(2) Association with mood and coping over time	(1) Wilcoxon test<br>(2) Actor–partner interdependence model (APIM)	EORTC QLQ-C30	Group	No

Drewes[37]	Observational cohort	2018	136	Unknown	42	Primary	(1) Compare mean scores between high grade and low grade glioma patients<br>(2) Compare two groups over time<br>(3) Explore clinical predictors for HRQoL	(1) Students T--test<br>(2) Mann-Whitney U test<br>(3) Logistic regression analyses	EQ-5D	Group	Yes
Lombardi[38]	Interventional Single-arm	2018	111	60 (median)	42	Primary	Compare two groups over time	Mixed-effect linear model	EORTC QLQ-C30, EORTC QLQ-BN20	Group	No
Wirsching[39]	Interventional randomized	2018	75	70 (median)	18	Secondary	(1) Compare two groups over time<br>(2) Deterioration-free survival	(1) Generalized linear mixed model<br>(2) Survival analysis	EORTC QLQ-C30, EORTC QLQ-BN20	Group	No
Liu[40]	Interventional non-randomized	2018	20	49 (median)	11	Primary	Compare HRQoL in one group over time at different time points	Mann-Whitney U test	EORTC QLQ-C30, EORTC QLQ-BN20	Group	Yes
Hall[41]	Interventional randomized	2019	427	Unknown	177	Primary	(1) Mean difference at various time points<br>(2) Compare two groups over time<br>(3) Effect of time from surgery to start of treatment	(1) Students T-test or Wilcoxon test<br>(2) Mixed effects models<br>(3) Linear regression model	EORTC QLQ-C30, EORTC QLQ-BN20	Group	No
Taphoorn[42]	Interventional randomized	2018	695	55 (mean)	202	Primary	(1) Differences between arms<br>(2) Compare two groups over time<br>(3) Percentage of patients with stable, improved or deteriorated HRQoL<br>(4) Deterioration-free survival and Time-to-deterioration	(1) Chi-square test, independent 2-tailed unpaired T-test, Mann-Whitney U test<br>(2) Linear mixed model repeated measures analyses	EORTC QLQ-C30, EORTC QLQ-BN20	Group and individual	Yes

								(3) Descriptive, area under the curve<br>(4) Survival analyses			
Wang[43]	Observational cohort	2018	260	42 (median)	155	Primary	Compare HRQoL in one group over time at different time points	Hierarchical linear models	FACT-Br	Group	No
Wick[44]	Interventional randomized	2017	437	58 (median)	172	Secondary	(1) Difference between arms at various time points<br>(2) Compare two groups over time<br>(3) Deterioration-free survival and Time-to-deterioration	(1) Unknown (2) Survival analyses	EORTC QLQ-C30, EORTC QLQ-BN20	Group	Yes
Schafer [45]	Interventional randomized	2018	170	56 (median)	56	Primary	(1) Difference between arms at various time points<br>(2) Compare 2 groups over time<br>(3) Deterioration-free survival and Time-to-deterioration	(1) Descriptive<br>(2) GEE models<br>(3) Survival analyses	EORTC QLQ-C30, EORTC QLQ-BN20	Group	Yes
Flechl[46]	Observational cohort	2017	42	54 (median)	15	Primary	Compare HRQoL in one group over time at different time points	Descriptive	EORTC QLQ-C30, EORTC EORTC QLQ-BN20	Group	No
Bitterlich[47]	Interventional non-randomized	2017	30	65 (median)	16	Primary	Compare HRQoL in one group over time at different time points	Mann-Whitney U test	EORTC QLQ-C30, EORTC QLQ-BN20	Group	No
Fariselli[48]	Interventional non-randomized	2017	35	54 (median)	14	Secondary	Compare HRQoL in one group over time at different time points	Students T-test	EORTC QLQ-C30, EORTC QLQ-BN20	Group	No
Randazzo[49]	Observational Cohort	2017	829	51 (median)	382	Primary	Association with psycho-social distress	Wilcoxon rank sum test	FACT-Br, FACT-F	Group	No

Diamond[50]	Observational Case-control	2017	50	50 (median)	16	Secondary	Association with prognostic awareness, prognostic communication and cognitive function	Chi-square test	FACT-Br	Group	No
Maschio[51]	Observational case-control	2017	25	Unknown	7	Secondary	Impact of HRQoL on mood and functional status at different time points	ANOVA	C30 and QOLIE 31-P	Group	No
Kaminska[52]	Interventional non-randomized	2017	15	49 (mean)	9	Primary	(1) Difference between patients and control group (2) Relation between HRQoL and perceived self-control	(1) Mann-Whitney U test (2) Spearman's rank correlation and regression analyses	WHOQOLBRE F	Group	No
Pollom[53]	Interventional non-randomized	2017	30	66 (median)	15	Primary	Compare HRQoL in one group over time at different time points	Mixed-effects linear regression models	EORTC QLQ-C30, EORTC QLQ-BN20 and MDASI-BT	Group	No
Field[54]	Interventional randomized	2017	117	55 (mean)	50	Primary	(1) Difference over time (2) Proportion of patients who improved/deteriorate d (3) Effect of treatment on proportion of deteriorating patients (4) Time-to-deterioration	(1) Descriptive (2) Logistic regression analyses (4) Survival analyses	EORTC QLQ-C30, EORTC QLQ-BN20	Group and individual	Yes
Bunevicius[55]	Observational cohort	2017	227	Unknown	Unkno wn	Primary	(1) Convergent validity (2) Comparison HRQoL scores with mood and functional status	(1) Spearman correlation coefficient (2) Mann-Whitney U test	SF-36	Group	No
Stockelmaier[56]	Observational cohort	2017	110	55 (mean)	51	Primary	(1) Association between progressive disease, molecular markers and HRQoL	(1) Spearman correlations (2) Linear mixed model	EORTC QLQ-C30, EORTC QLQ-BN20	Group	No

							(2) Impact of clinical and treatment variables on HRQoL				
Gangliardi[57]	Observational cohort	2017	39	Unknown	21	Secondary	(1) Differences between mean scores before and after treatment<br>(2) Comparison with norm group<br>(3) Prognostic model (HRQoL outcome)	(1) Students T-test, Mann-Whitney U test<br>(2) Students T-test, Mann-Whitney U test<br>(3) Cox proportional Hazard model	SF-36	Group	No
Culos-Reed[58]	Interventional non-randomized	2017	15	51 (mean)	7	Secondary	Associations between fitness, clinical variables and HRQoL	Spearman's Rho	FACT-Br	Group	No
Kim[59]	Observational cohort	2016	81	44	26	Primary	(1) Differences in clinical variables between glioma and meningioma<br>(2) Relation between the symptoms<br>(3) Identify predictors for HRQoL	(1) Chi-square test, Fisher exact test, independent T- test or Mann-Whitney U test<br>(2) Principal components analyses<br>(3) Hierarchical multiple regression analyses	MDASI-BT	Group	No
Ediebah[60]	Cross-sectional	2017	195	41	75	Primary	(1) Difference in patient and proxy scores<br>(2) Proportion of patients whose score differed between patients and proxies<br>(3) Agreement between patient and proxy scores	(1) Wilcoxon signed-rank test<br>(2) Descriptive<br>(3) Bland–Altman limits of agreement	SF-36, EORTC QLQ-BN20	Group and individual	No

Reijneveld[61]	Interventional randomized	2016	477	44 (mean)	202	Secondary	(1) Difference in HRQoL at specific timepoints<br>(2) Difference in HRQoL over time<br>(3) Change from baseline	(1) Area under the curve<br>(2) Linear mixed models<br>(3) Survival analyses	EORTC QLQ-C30, EORTC QLQ-BN20	Group and individual	Yes
Baumstarck[62]	Cross-sectional	2016	17	58 (mean)	42	Primary	(1) Comparison between patients and caregivers<br>(2) Association between HRQoL and coping	(1) Wilcoxon test<br>(2) Correlation analyses<br>(2) Actor–partner interdependence model	Patient-Generated Index (PGI), EORTC QLQC30	Group	No
Paquette[63]	Observational cohort	2016	134	Unknown	54	Primary	(1) Comparison of mean scores<br>(2) Prediction model (HRQoL is outcome)	(1) Student's T-test, chi-square test<br>(2) Multivariable Cox proportional hazard model, C-index	EORTC QLQ-C30, EORTC QLQ-BN20	Group	No
Wolf[64]	Observational cohort	2016	22	55 (median)	8	Primary	(1) Difference in HRQoL between two timepoints	(1) Reliable change index	EORTC QLQ-C30, EORTC QLQ-BN20	Group	No
Bigatao[65]	Interventional non-randomized	2015	22	Unknown	Unknown	Secondary	(1) Comparison between two groups<br>(2) Comparison at different time points	(1) Mixed-effects regression model<br>(2) Mixed-effects regression model	EORTC QLQ-C30, EORTC QLQ-BN20	Group	No
Mahalakshmi[66]	Observational cohort	2019	162	Unknown	64	Primary	(1) Differences at baseline between groups<br>(2) Association between clinical and treatment variables and HRQoL	(1)Mann Whitney U-test, Kruskal-Wallis Test<br>(2) Spearman's rank correlations	EORTC QLQ-C30, EORTC QLQ-BN20	Group	Yes

Lee[67]	Interventional randomized	2016	81	Unknown	36	Secondary	Difference between groups	Unknown	FACT-G	Group	No
Suchorska[68]	Interventional randomized	2016	71	Unknown	36	Secondary	Comparison between two groups	Mann–Whitney U test	EORTC QLQ-C30, EORTC QLQ-BN20	Group	No
Noll[69]	Observational cohort	2018	103	52	43	Primary	(1) Association between HRQoL and neurocognitive functioning (2) Identifying predictors for HRQoL	(1) Pearson correlations (2) Stepwise multiple regression analyses	FACT-G	Group	Yes
Odia[70]	Interventional cohort	2016	80	51	34	Secondary	Proportion of patients with a meaningful change in HRQoL	Descriptive	FACT-Br	Individual	Yes
Lucchiari[71]	Observational cohort	2015	85	48 (mean)	39	Primary	(1) Association between clinical variables and HRQoL (2) Compare at two timepoints	(1) Pearson's correlations and linear regression analyses (2) Paired T-tests	FACT-Br	Group	
Sagberg[72]	Interventional non-randomized	2016	30	62 (mean)	7	Primary	(1) Calculate cumulative HRQoL in the first year (2) Variables associated with HRQoL	(1) Area under the curve (2) Linear regression analyses	EQ-5D 3L	Group	Yes
Edelstein[73]	Observational cohort	2016	73	53 (mean)	29	Secondary	(1) Compare group means to different norm groups (2) Association between coping mechanisms and HRQoL	(1) ANOVA (2) Hierarchical multiple regression analyses	EORTC QLQ-C30, EORTC QLQ-BN20	Group	No
Armstrong[74]	Cross-sectional	2016	617	47 (median)	251	Primary	(1) Prevalence of symptoms (2) Subgroup comparisons of symptoms	(1) Descriptive (2) Chi-square tests	MDASI-BT	Group	No

Okita[75]	Observational cohort	2015	50	39 (median)	16	Primary	Assess independent risk factors for HRQoL	Multivariate analysis, multiple regression analyses	EORTC QLQ-C30, EORTC QLQ-BN20	Group	Yes
Jakola[76]	Interventional non-randomized	2015	22	67 (median)	15	Primary	(1) Compare HRQoL before and after surgery (2) Proportion of patients with a meaningful change in HRQoL	(1) Paired samples T-test (2) Descriptive	EQ-5D	Group and individual	Yes
Oberg[77]	Interventional non-randomized	2014	16	50 (median)	5	Secondary	(1) Proportion of patients with a meaningful change in HRQoL (2) Associations between outcome variables	(1) Descriptive (2) Pearson correlations	SF-36	Group and individual	No
Armstrong[78]	Observational cohort	2014	100	48 (mean)	35	Primary	Proportion of patients who reported symptoms	Descriptive	MDASI-BT	Individual	No
Jacobs[79]	Cross-sectional	2014	45	53 (median)	18	Primary	(1) Degree of chance-corrected agreement between patients and caregivers (2) Association between patient and proxy scores	(1) Intraclass correlation coefficients (2) Linear mixed-effects models	FACT-Br	Group	No
Boele[80]	Observational cohort	2014	195	41 (median)	77	Primary	(1) Comparison between patients and caregivers (2) Association between cognitive functioning and HRQoL	(1) ANOVA and chi-square (2) Pearson correlations	EORTC QLQ-C30, EORTC QLQ-BN20	Group	No
Piil[81]	Observational cohort	2015	30	58 (median)	11	Primary	(1) Difference between baseline and follow-up scores	(1) Paired T-tests (2) Linear mixed models	FACT-Br	Group	No

							(2) Association between HRQoL, mood and clinical characteristics				
Taphoorn[82]	Interventional randomized	2015	902	Unknown	335	Primary	(1) Mean change from baseline<br>(2) Deterioration-free survival and Time-to-deterioration	(1) Linear mixed model<br>(2) Survival analyses	EORTC QLQ-C30, EORTC QLQ-BN20	Group and individual	Yes
Dirven[83]	Interventional randomized	2015	138	Unknown	55	Primary	(1) Change over time<br>(2) Comparison with norm group<br>(3) Proportion of patients with a meaningful change in HRQoL	(1) Survival analyses<br>(2) Unknown<br>(3) Descriptive analyses	EORTC QLQ-C30, EORTC QLQ-BN20	Group and individual	Yes
Lucchiari[84]	Cross-sectional	2015	73	49 (mean)	25	Secondary	(1) Association between HRQoL and clinical variables<br>(2) Association between HRQoL and other outcomes<br>(3) Inter-rate reliability	(1) Spearman rho coefficient<br>(2) Linear regression analyses<br>(3) Krippendorff's alpha coëfficiënt	FACT-Br	Group	No
Aprile[85]	Cross-sectional	2015	67	53 (mean)	24	Secondary	Predictors of fatigue (HRQoL as predictor)	Multiple linear regression analyses	EORTC QLQ-C30, EORTC QLQ-BN20	Group	No
Boele[86]	Observational cohort	2015	65	45 (mean)	29	Primary	(1) Comparison with norm group<br>(2) Difference between time points<br>(3) Proportion of patients with a meaningful change in HRQoL	(1) Independent sample T-tests<br>(2) Wilcoxon signed rank tests<br>(3) Descriptive	SF-36	Group and individual	No
Halkett[87]	Observational cohort	2015	116	Unknown	34	Primary	Association with other variables (distress, needs)	Correlation analyses, linear and logistic regression and	FACT-Br, FACT-G	Group	No

								multivariable linear models			
Westphal[88]	Interventional randomized	2015	142	Unknown	55	Secondary	Compare mean scores between groups	Descriptive	EORTC QLQ-C30, EORTC QLQ-BN20	Group	No
Shih[89]	Interventional non-randomized	2014	20	38 (median)	7	Secondary	Analyses of HRQoL over time	Linear mixed models	FACT-Br, FACT-G	Group	No
Ahluwalia[90]	Interventional non-randomized	2015	62	43 (median)	30	Secondary	Difference between two time points	Wilcoxon signed-rank test	EORTC QLQ-C30, EORTC QLQ-BN20	Group	No
Osman[91]	Interventional non-randomized	2014	29	42 (mean)	9	Secondary	Proportion of patients with a meaningful change in HRQoL	Descriptive	FACT-15 (FBrSI-15)	Group	Yes
Ediebah[92]	Interventional randomized	2014	288	Unknown	156	Primary	Analyses of HRQoL over time	Joint model	EORTC QLQ-C30, EORTC QLQ-BN20	Group	Yes
Peters[93]	Observational cohort	2015	237	50 (mean)	112	Primary	Prediction model (HRQoL is predictor)	Cox proportional hazard model	FACT-Br, FACT-G	Group	No
Osman[94]	Interventional non-randomized	2014	20	29 (mean)	5	Secondary	Proportion of patients with a meaningful change in HRQoL	Descriptive	FACT-15 (FBrSI-15)	Group	Yes
Jakola[95]	Observational cohort	2014	79	43 (mean)	38	Primary	Comparison of means between groups	Independent samples T-test	EQ-5D, EORTC QLQ-C30, EORTC QLQ-BN20	Group	No
Giovagnoli[96]	Observational case-control	2014	291	43 (mean)	113	Primary	(1) Comparison between five patient groups and controls (2) Association between HRQoL and clinical and treatment variables	(1) ANOVA (2) Multiple stepwise regression analyses	Functional Living Index-Cancer (FLIC)	Group	No
Chinot[97]	Interventional randomized	2014	921	57 (mean)	341	Secondary	Deterioration free survival and Time-to-deterioration	Survival analyses	EORTC QLQ-C30, EORTC QLQ-BN20	Group	Yes

Gilbert[98]	Interventional randomized	2014	637	Unknown	258	Secondary	Difference in changes between the two groups	General linear models	EORTC QLQ-C30, EORTC QLQ-BN20	Group	Yes
Porter[99]	Cross-sectional	2015	26	58 (median)	13	Primary	(1) Association between FACT-Br and FPQLI-C scales (2) Compare mean HRQoL scores and patient factors	(1) Pearson correlation (2) Independent samples T-test	FPQLI-C, FACT-Br	Group	No
Sagberg[100]	Observational cohort	2014	164	56 (mean)	75	Primary	(1) Association between HRQoL and performance status (2) Differences in pre and post-operative HRQoL	(1) Spearman correlation (2) Paired samples T-test	EG-5D 3L	Group	Yes
Hutterer[101]	Interventional non-randomized	2013	40	58 (median)	11	Secondary	Difference in HRQoL between treatment arms over time	Linear mixed models	EORTC QLQ-C30, EORTC QLQ-BN20	Group	No
Habets[102]	Observational cohort	2014	32	57 (mean)	13	Primary	(1) HRQoL over time (2) Difference between patients and healthy controls (3) Associations between HRQoL and clinical and cognitive variables	(1) Wilcoxon signed rank test (2) Kolmogorov–Smirnov test (3) Spearman's rho , Mann–Whitney U tests	EORTC QLQ-C30, EORTC QLQ-BN20	Group	Yes
Armstrong[103]	Observational cohort	2013	182	Unknown	88	Primary	(1) Differences between arms (2) Prediction model (HRQoL is predictor of survival)	(1) Two-sample proportion tests (2) Cox proportional hazard model	EORTC QLQ-C30, EORTC QLQ-BN20 and MDASI-BR	Group	Yes
Yu[104]	Interventional randomized	2013	58	Unknown	Unknown	Secondary	Compare variables between the three groups	Chi-square test, Students t-test, ANOVA	EORTC QLQ-C30, EORTC QLQ-BN20	Group	No
Daigle[105]	Observational cohort	2013	35	Unknown	13	Primary	(1) Association between two time points (2) Association between HRQoL,	(1) Wilcoxon signed rank tests (2) Spearman correlations,	SNAS	Group	No

							tumor volume and resection<br>(3) Prediction model (HRQoL is predictor of survival)	linear regression analyses<br>(3) Cox proportional hazard model			
Reddy[106]	Interventional non-randomized	2013	24	61 (median)	10	Primary	Difference in mean HRQoL scores between groups	Descriptive	EORTC QLQ-C30, EORTC QLQ-BN20	Group	Yes
Cabrera[107]	Interventional non-randomized	2013	15	53 (median)	3	Secondary	Compare mean scores over time	Descriptive	FACT-Br	Group	No
Minniti[108]	Interventional randomized	2013	65	73 (median)	32	Primary	(1) Change scores between the two treatment arms over time<br>(2) Proportion of patients with a meaningful change	(1) Students T-test<br>(2) Descriptive	EORTC QLQ-C30, EORTC QLQ-BN20	Group and individual	Yes
Jakola[109]	Observational cohort	2012	55	41 (mean)	25	Primary	(1) Compare HRQoL between groups<br>(2) Prediction models (HRQoL predictor of survival)	(1) Independent samples T-test<br>(2) Cox proportional hazard model	EORTC QLQ-C30, EORTC QLQ-BN20 and EQ5D	Group	No
Malmstrom[110]	Interventional randomized	2012	284	Unknown	Unknown	Secondary	Changes in mean HRQoL for each group	Kruskal-Wallis test and pairwise comparisons With, Mann-Whitney U test	EORTC QLQ-C30, EORTC QLQ-BN20	Group	No
Stupp[111]	Interventional randomized	2012	120	54 (median)	28	Secondary	Change in HRQoL from baseline	Descriptive	EORTC QLQ-C30, EORTC QLQ-BN20	Group	No
Jakola[112]	Observational cohort	2011	67	58 (mean)	29	Primary	(1) Compare HRQoL before and after surgery<br>(2) Prediction model (HRQoL is predictor of survival)	(1) Paired sample t-test, TMann-Whitney U test<br>(2) Cox proportional hazard model	EQ-5D 3L	Group	No

Yavas[113]	Observational cohort	2012	118	Unknown	45	Primary	(1) Compare HRQoL at different time points (2) Compare groups	(1) Wilcoxon test (2) Mann–Whitney U test	EORTC QLQ-C30, EORTC QLQ-BN20	Group	No
Aaronson[114]	Observational case-control	2011	195	41 (mean)	39	Primary	(1) Compare HRQoL between groups (2) Association between clinical and treatment variables and HRQoL	(1) ANOVA's (2) Multivariable linear regression analyses	SF-36 and EORTC QLQ-BN20	Group	No
Yavas[115]	Observational cohort	2012	39	Unknown	16	Primary	(1) Compare HRQoL at different time points (2) Compare groups	(1) Wilcoxon test (2) Mann–Whitney U test	EORTC QLQ-C30, EORTC QLQ-BN20	Group	No
Gehring[116]	Interventional randomized	2012	24	Unknown	11	Secondary	(1) Baseline difference between groups (2) Group mean change over time (3) Difference in proportion of patients with change score	(1) Independent samples T-test (2) Students T-tests (3) Fisher's exact tests	FACT-G, FACT-Br	Group	No
Ruiz[117]	Interventional non-randomized	2012	18	50 (median)	7	Secondary	(1) Differences in baseline scores (2) Differences between groups over time	(1) Descriptive	FACT-Br	Group	No
Maschio[118]	Observational cohort	2012	25	50 (mean)	11	Secondary	Difference in HRQoL scores at different time points	Wilcoxon test	EORTC QLQ-C30, QOLIE-31P V-2	Group	No
Blonski[119]	Observational cohort	2012	10	Unknown	1	Secondary	Comparison with normgroup	Descriptive	EORTC QLQ-C30, EORTC QLQ-BN20	Group	No
Gallego Perez-Larraya[120]	Interventional non-randomized	2011	70	77 (median)	42	Secondary	Change from baseline	Linear mixed model	EORTC QLQ-C30, EORTC QLQ-BN20	Group	No
Jakola[121]	Observational cohort	2011	88	57 (mean)	38	Primary	(1) Compare HRQoL between subgroups (2) Compare HRQoL before and after surgery	(1) Mann-Whitney U, Kruskal-Wallis test	EQ-5D 3L	Group	No

							(3) Prediction model (HRQoL is outcome)<br>(4) Correlations between different outcome variables	(2) Wilcoxon signed-rank test<br>(3) Multivariate regression model<br>(4) Spearman rho			
Armstrong[122]	Cross-sectional	2011	294	46 (median)	119	Primary	Prediction model (symptoms as predictor for progression)	Multivariable logistic regression model	MDASI	Group	Yes
Brada[123]	Interventional randomized	2010	447	Unknown	159	Secondary	Proportion of patients with a meaningful change in HRQoL	Descriptive	EORTC QLQ-C30, EORTC QLQ-BN20	Group	Yes
Ruge[124]	Cross-sectional	2011	33	42 (median)	17	Primary	(1) Comparison with normgroup<br>(2) Proportion of patients with scores below the normgroup<br>(3) Compare HRQoL between subgroups	(1) T-values<br>(2) Descriptive<br>(3) Mann–Whitney U tests	SF-36 and EORTC QLQ-BN20	Group and individual	No
Terasaki[125]	Interventional non-randomized	2011	26	61 (median)	9	Secondary	(1) Comparison of mean scores over time<br>(2) Comparison of HRQoL with performance score	(1) Unknown	FACT-Br	Group	No
Cheng[126]	Cross-sectional	2010	92	42 (median)	35	Primary	(1) Differences between subgroups<br>(2) Association HRQoL with clinical variables	(1) Mann Whitney U-test or Kruskal-Wallis test<br>(2) Spearman's rank correlation	EORTC QLQ-C30	Group	No
Jones[127]	Observational cohort	2010	25	47 (mean)	14	Secondary	(1) Comparison of baseline scores between two groups<br>(2) Change over time between groups<br>(3) Association between different endpoints	(1) Independent-samples T-tests, Pearson's tests<br>(2) ANOVA's<br>(3) Linear regression analyses	FACT-Br	Group	No

Kong [128]	Observational cohort	2010	38	51 (median)	16	Secondary	Difference over time	Unknown	SF-36	Group	No
Lucchiari[129]	Cross-sectional	2010	84	Unknown	37	Primary	Compare HRQoL between subgroups	ANOVA's	FACT-Br	Group	No
Jones[130]	Cross-sectional	2010	35	47 (mean)	14	Secondary	(1) Differences between low grade and high grade patients (2) Association between study endpoints	(1) Independent samples T-test (2) Linear regression analyses	FACT-Br	Group	No
Mobed[131]	Cross-sectional	2009	718	Unknown	295	Primary	(1) Compare HRQoL between subgroups (2) Association between outcomes	(1) Chi-square tests, two-sample T-tests (2) Multiple logistic regression analyses	FACT-Br	Group	No
Wang[132]	Interventional randomized	2010	289	Unknown	Unknown	Primary	(1) Group differences at baseline (2) Assessment of HRQoL, cognitive functioning and survival over time (3) prediction model (HRQoL is outcome)	(1) Chi-square tests (2) Joint modeling (3) Unknown	BQOL	Group	No
Edvardsson[133]	Observational case-control	2009	39	Unknown	12	Primary	Difference in HRQoL between subgroups	Mann-Whitney U test	SQOL	Group	No
Corn[134]	Interventional randomized	2009	185	Unknown	Unknown	Primary	Differences from baseline to Follow up	Wilcoxon signed rank sum test	Spitzer Quality of Life Index	Group	No
Gehring[135]	Interventional randomized	2009	140	Unknown	59	Secondary	(1) Compare baseline characteristics between groups (2) Differences over time between groups	(2) Independent T-tests, Mann-Whitney tests were (2) ANCOVA's	SF-36	Group	No
Kvale[136]	Cross-sectional	2009	50	53 (mean)	21	Primary	(1) Compare	(1) Wilcoxon rank sum test	FACT-Br	Group	No

							distress and quality of life scores by race and gender<br>(2) Association between distress and HRQoL	(2) Spearman rank correlation coefficients, chi-square test			
Jones[137]	Cross-sectional	2009	171	50 (mean)	54	Secondary	Association between various outcomes	Linear regression analysis	FACT-Br	Group	No
Kocher[138]	Interventional randomized	2008	62	Unknown	21	Secondary	Comparison of HRQoL over time between groups	Descriptive	EORTC QLQ-C30, EORTC QLQ-BN20	Group	No
Liu[139]	Interventional non-randomized	2009	66	Unknown	26	Primary	(1) Differences between subgroups<br>(2) Correlation between age and HRQoL<br>(3) Comparison with normgroup<br>(4) Comparison follow-up and baseline scores<br>(5) Change over time controlled for clinical variables	(1) Kruskal-Wallis test<br>(2) Kendall rank correlation coefficient<br>(3) Two-sample T-test<br>(4) Two-sample T-test<br>(5) Hierarchical linear models	FACT-Br	Group	Yes
Bosma[140]	Observational cohort	2009	32	Unknown	Unknown	Primary	(1) Difference between groups<br>(2) Difference between baseline and follow-up scores	(1) ANCOVA's<br>(2) Wilcoxon signed-rank test	SF-36 and EORTC QLQ-BN20	Group	Yes
Brown[141]	Observational cohort	2008	197	55 (mean)	124	Primary	(1) Compare HRQoL scores between patients and caregivers<br>(2) Proportion of patients with a clinically relevant change score	(1) Paired signed rank test<br>(2) Descriptive<br>(3) Spearman's correlation<br>(4) Regression model	FACT-Br	Group	Yes

							(3) Association between patient and caregiver scores<br>(4) Association between HRQoL and MMSE				
Mauer[142]	Interventional randomized	2007	247	Unknown	Unknown	Primary	Predictive models (HRQoL as predictor for survival)	Cox proportional hazard models, C-index	EORTC QLQ-C30, EORTC QLQ-BN20	Group	Yes
Taphoorn[143]	Interventional randomized	2007	288	Unknown	Unknown	Secondary	(1) Differences between the treatment groups over time<br>(2) Proportion of patients with a clinically relevant change score	(1) Mixed model<br>(2) Descriptive analyses	EORTC QLQ-C30, EORTC QLQ-BN20	Group	Yes
Rabbani[144]	Interventional non-randomized	2007	23	58 (mean)	9	Secondary	Differences between the treatment groups over time	Unknown	EORTC QLQ-C30, EORTC QLQ-BN20	Group	No
Mauer[145]	Interventional randomized	2007	490	Unknown	Unknown	Primary	Predictive models (HRQoL as predictor for survival)	Cox proportional hazard models, C-index	EORTC QLQ-C30, EORTC QLQ-BN20	Group	Yes
Keime-Guibert[146]	Interventional randomized	2009	209	Unknown	Unknown	Secondary	(1) Difference in HRQoL from baseline to follow-up timepoints<br>(2) Difference between baseline and follow-up assessments	(1) Wilcoxon signed rank test<br>(2) Reliable change index	Spitzer Quality of Life Index	Group	No
Ernst-Stecken[147]	Observational cohort	2007	10	49 (median)	Unknown	Secondary	Difference in HRQoL from baseline to follow-up timepoints	Unknown	EORTC QLQ-C30, EORTC QLQ-BN20	Group	No
Gustafsson[148]	Cross-sectional	2006	39	47 (mean)	12	Primary	(1) Describe HRQoL<br>(2) Investigate relations between function, HRQoL and coping	(1) Descriptive<br>(2) Spearman's rank correlation	EORTC QLQ-C30	Group	No

Levin[149]	Interventional randomized	2006	162	Unknown	54	Secondary	Difference between groups over time	Unknown	FACT-Br	Group	No
Henriksson[150]	Interventional randomized	2006	140	Unknown	40	Secondary	Difference between groups over time	Unknown	EORTC QLQ-C30	Group	No
Taphoorn[151]	Interventional randomized	2005	573	185	Unknown	Primary	Difference between groups over time	Mixed-model approach	EORTC QLQ-C30, EORTC QLQ-BN20	Group	No
Brown[152]	Observational cohort	2006	220	67	57	Primary	(1) Difference between groups<br>(2) Association between HRQoL and disease variables<br>(3) Associations between HRQoL and disease variables<br>(4) Prognostic model (HRQoL as predictor for survival)	(1) Kruskal-Wallis test, Wilcoxon rank sum test<br>(2) Spearman rank correlation coefficient<br>(3) Kruskal-Wallis test, Wilcoxon rank sum test<br>(4) Cox proportional hazards models	FACT-Br	Group	No
Tuettenberg[153]	Interventional non-randomized	2004	13	Unknown	5	Secondary	Comparing HRQoL results during therapy	Unknown	EORTC QLQ-C30	Group	No
Souhami[154]	Interventional randomized	2004	203	Unknown	75	Secondary	Comparing HRQoL results during treatment	Unknown	Spitzer Quality of Life Index	Group	No
Roa[155]	Interventional randomized	2004	100	Unknown	40	Secondary	Comparing HRQoL results during treatment	Unknown	FACT-Br	Group	No
Brada[156]	Observational case-control	2003	30	40 (median)	17	Secondary	Proportion of patients with change score	Descriptive	EORTC QLQ-C30	Individual	Yes
Klein[157]	Observational case-control	2003	239	Unknown	Unknown	Primary	(1) Differences between groups<br>(2) Association with epilepsy and cognitive functioning	(1) ANCOVA<br>(2) Stepwise linear regression analyses	SF-36	Group	No
Walker[158]	Observational cohort	2003	159	Unknown	92	Primary	(1) Association with survival	(1) Univariable survival analyses<br>(2) Descriptive	EORTC QLQ-C30, EORTC QLQ-BN20	Group	No

							(2) Compare HRQoL results over time				
Prados[159]	Interventional randomized	2003	122	Unknown	40	Secondary	Difference in HRQoL between groups over time	Unknown	FACT-Br	Group	No
Khan[160]	Interventional non-randomized	2002	35	Unknown	10	Secondary	Compare mean scores over time	Paired sampled T-test	FACT-Br	Group	No
Brada[161]	Interventional non-randomized	2001	138	Unknown	53	Secondary	Proportion of patients with change score	Descriptive	EORTC QLQ-C30, EORTC QLQ-BN20	Individual	Yes
Osoba[162]	Interventional randomized	2000	unknown	Unknown	Unknown	Primary	(1) Compare mean scores over time<br>(2) Proportion of patients with symptoms	(1) Descriptive	EORTC QLQ-C30, EORTC QLQ-BN20	Group and individual	No
Reijneveld[163]	Cross-sectional	2001	48	Unknown	23	Primary	(1) Differences between patients and controls<br>(2) Difference between patient groups	(1) Kruskal–Wallis test<br>(2) Mann–Whitney U test	SF-36	Group and individual	No
Bampoe[164]	Interventional randomized	2000	129	Unknown	Unknown	Primary	(1) Difference at specific moments during follow-up<br>(2) Association between performance status and HRQoL	(1) Wilcoxon rank-sum test<br>(2) Spearman correlation coefficients	EORTC QLQ-C30, EORTC QLQ-BN20	Group	No
Osoba[165]	Cross-sectional	2000	162	43 (mean)	60	Primary	Proportion of patients with change score	Descriptive	EORTC QLQ-C30, EORTC QLQ-BN20	Individual	yes
Yung[166]	Interventional non-randomized	2000	225	Unknown	Unknown	Secondary	Proportion of patients with change score	Descriptive	EORTC QLQ-C30, EORTC QLQ-BN20	Individual	No
Postma[167]	Interventional non-randomized	2000	17	Unknown	3	Secondary	Comparison with normgroup	Unknown	EORTC QLQ-C30	Individual	No

Osoba[168]	Interventional non-randomized	2000	179	Unknown	61	Primary	(1) Change from baseline<br>(2) Difference in mean scores between groups	(1) Descriptive<br>(2) Students T-tests	EORTC QLQ-C30, EORTC QLQ-BN20	Group	Yes
Yung[169]	Interventional non-randomized	1999	162	Unknown	69	Primary	Proportion of patients with change score	Descriptive	EORTC QLQ-C30, EORTC QLQ-BN20	Group	No
Powell[170]	Interventional non-randomized	2011	68	47 (median)	18	Secondary	(1) Change from baseline<br>(2) Difference between groups	(1) Paired T-test<br>(2) ANOVA	EORTC QLQ-C30, EORTC QLQ-BN20	Group	No

EORTC: European organization of research and treatment of cancer. MCID: minimally clinical important difference

References

1. Piil, K.; Christensen, I.J.; Grunnet, K.; Poulsen, H.S. Health-related quality of life and caregiver perspectives in glioblastoma survivors: a mixed-methods study. *BMJ Support Palliat Care* **2019**, 10.1136/bmjspcare-2019-001777, doi:10.1136/bmjspcare-2019-001777.
2. Haraldseide, L.M.; Jakola, A.S.; Solheim, O.; Sagberg, L.M. Does preoperative health-related quality of life predict survival in high-grade glioma patients? - a prospective study. *Br J Neurosurg* **2020**, *34*, 28-34, doi:10.1080/02688697.2019.1698011.
3. Vaitkiene, P.; Pranckeviciene, A.; Stakaitis, R.; Steponaitis, G.; Tamasauskas, A.; Bunevicius, A. Association of miR-34a Expression with Quality of Life of Glioblastoma Patients: A Prospective Study. *Cancers (Basel)* **2019**, *11*, doi:10.3390/cancers11030300.
4. Mukherjee, S.; Wood, J.; Liaquat, I.; Stapleton, S.R.; Martin, A.J. Craniotomy for recurrent glioblastoma: Is it justified? A comparative cohort study with outcomes over 10 years. *Clinical neurology and neurosurgery* **2020**, *188*, 105568.
5. Randazzo, D.M.; McSherry, F.; Herndon, J.E.; Affronti, M.L.; Lipp, E.S.; Flahiff, C.; Miller, E.; Woodring, S.; Boulton, S.; Desjardins, A. Complementary and integrative health interventions and their association with health-related quality of life in the primary brain tumor population. *Complementary therapies in clinical practice* **2019**, *36*, 43-48.
6. Hansen, A.; Pedersen, C.B.; Minet, L.R.; Beier, D.; Jarden, J.O.; Søgaard, K. Hemispheric tumor location and the impact on health-related quality of life, symptomatology, and functional performance outcomes in patients with glioma: an exploratory cross-sectional study. *Disability and rehabilitation* **2019**, 1-7.
7. Coomans, M.; Dirven, L.; Aaronson, N.K.; Baumert, B.G.; van den Bent, M.; Bottomley, A.; Brandes, A.A.; Chinot, O.; Coens, C.; Gorlia, T. The added value of health-related quality of life as a prognostic indicator of overall survival and progression-free survival in glioma patients: a meta-analysis based on individual patient data from randomised controlled trials. *European Journal of Cancer* **2019**, *116*, 190-198.
8. Onken, J.S.; Goerling, U.; Heinrich, M.; Pleissner, S.; Krex, D.; Vajkoczy, P.; Misch, M. Patient Reported Outcome (PRO) Among High-Grade Glioma Patients Receiving TTFields Treatment: A Two Center Observational Study. *Frontiers in neurology* **2019**, *10*, 1026.
9. Renovanz, M.; Maurer, D.; Lahr, H.; Weimann, E.; Deininger, M.; Wirtz, C.R.; Ringel, F.; Singer, S.; Coburger, J. Supportive Care Needs in Glioma Patients and Their Caregivers in Clinical Practice: Results of a Multicenter Cross-Sectional Study. *Front Neurol* **2018**, *9*, 763, doi:10.3389/fneur.2018.00763.
10. Gabel, N.; Altshuler, D.B.; Brezzell, A.; Briceño, E.M.; Boileau, N.R.; Miklja, Z.; Kluin, K.; Ferguson, T.; McMurray, K.; Wang, L., et al. Health Related Quality of Life in Adult Low and High-Grade Glioma Patients Using the National Institutes of Health Patient Reported Outcomes Measurement Information System (PROMIS) and Neuro-QOL Assessments. *Front Neurol* **2019**, *10*, 212, doi:10.3389/fneur.2019.00212.
11. Li, J.; Sun, L.; Wang, X.; Sun, C.; Heng, S.; Hu, X.; Chen, W.; Liu, F. Are Posttraumatic Stress Symptoms and Avoidant Coping Inhibitory Factors? The Association Between Posttraumatic Growth and Quality of Life Among Low-Grade Gliomas Patients in China. *Front Psychol* **2019**, *10*, 330, doi:10.3389/fpsyg.2019.00330.
12. Dirven, L.; Luerding, R.; Beier, D.; Bumes, E.; Reinert, C.; Seidel, C.; Bonsanto, M.M.; Bremer, M.; Rieken, S.; Combs, S.E., et al. Neurocognitive functioning and health-related quality of life in adult medulloblastoma patients: long-term outcomes of the NOA-07 study. *Journal of Neuro-Oncology* **2020**, *148*, 117-130, doi:10.1007/s11060-020-03502-y.
13. Scartoni, D.; Amelio, D.; Palumbo, P.; Giacomelli, I.; Amichetti, M. Proton therapy re-irradiation preserves health-related quality of life in large recurrent glioblastoma. *Journal of Cancer Research and Clinical Oncology* **2020**, *146*, 1615-1622, doi:10.1007/s00432-020-03187-w.
14. Faarup, I.; Lauridsen, J.T.; Lütgen, K.; Nørregaard, A.; Poulsen, F.R.; Østergaard, B. Do family health conversations impact patients with glioblastoma multiforme and their family members? *J Clin Nurs* **2019**, *28*, 1695-1707, doi:10.1111/jocn.14777.
15. Wick, W.; Krendyukov, A.; Junge, K.; Höger, T.; Fricke, H. Longitudinal analysis of quality of life following treatment with Asunercept plus reirradiation versus reirradiation in progressive glioblastoma patients. *J Neurooncol* **2019**, *145*, 531-540, doi:10.1007/s11060-019-03320-x.
16. Dirven, L.; Reijneveld, J.C.; Taphoorn, M.J.B.; Coens, C.; El-Badawy, S.A.; Tzuk-Shina, T.; Bravo-Marques, J.; Back, M.; Stalpers, L.J.A.; Stupp, R., et al. Impact of Radiation Target Volume on Health-Related Quality of Life in Patients With Low-Grade Glioma in the 2-Year Period Post Treatment: A Secondary Analysis of the EORTC 22033-26033. *Int J Radiat Oncol Biol Phys* **2019**, *104*, 90-100, doi:10.1016/j.ijrobp.2019.01.003.
17. Bø, H.K.; Solheim, O.; Kvistad, K.A.; Berntsen, E.M.; Torp, S.H.; Skjulsvik, A.J.; Reinertsen, I.; Iversen, D.H.; Unsgård, G.; Jakola, A.S. Intraoperative 3D ultrasound-guided resection of

regarding health-related quality of life in low-grade glioma patients. *Qual Life Res* **2017**, *26*, 869-880, doi:10.1007/s11136-016-1426-z [doi];10.1007/s11136-016-1426-z [pii].
61. Reijneveld, J.C.; Taphoorn, M.J.; Coens, C.; Bromberg, J.E.; Mason, W.P.; Hoang-Xuan, K.; Ryan, G.; Hassel, M.B.; Enting, R.H.; Brandes, A.A., et al. Health-related quality of life in patients with high-risk low-grade glioma (EORTC 22033-26033): a randomised, open-label, phase 3 intergroup study. *Lancet Oncol* **2016**, *17*, 1533-1542, doi:S1470-2045(16)30305-9 [pii];10.1016/S1470-2045(16)30305-9 [doi].
62. Baumstarck, K.; Leroy, T.; Hamidou, Z.; Tabouret, E.; Farina, P.; Barrie, M.; Campello, C.; Petrirena, G.; Chinot, O.; Auquier, P. Coping with a newly diagnosed high-grade glioma: patient-caregiver dyad effects on quality of life. *J Neurooncol* **2016**, *129*, 155-164, doi:10.1007/s11060-016-2161-6 [doi];10.1007/s11060-016-2161-6 [pii].
63. Paquette, B.; Vernerey, D.; Chauffert, B.; Dabakuyo, S.; Feuvret, L.; Taillandier, L.; Frappaz, D.; Taillia, H.; Schott, R.; Ducray, F., et al. Prognostic value of health-related quality of life for death risk stratification in patients with unresectable glioblastoma. *Cancer Med* **2016**, *5*, 1753-1764, doi:10.1002/cam4.734 [doi].
64. Wolf, J.; Campos, B.; Bruckner, T.; Vogt, L.; Unterberg, A.; Ahmadi, R. Evaluation of neuropsychological outcome and "quality of life" after glioma surgery. *Langenbecks Arch Surg* **2016**, *401*, 541-549, doi:10.1007/s00423-016-1403-6 [doi];10.1007/s00423-016-1403-6 [pii].
65. Bigatao, M.R.; Peria, F.M.; Tirapelli, D.P.; Carlotti Junior, C.G. Educational program on fatigue for brain tumor patients: possibility strategy? *Arq Neuropsiquiatr* **2016**, *74*, 155-160, doi:S0004-282X2016000200014 [pii];10.1590/0004-282X20160007 [doi].
66. Mahalakshmi, P.; Vanisree, A.J. Quality of life measures in glioma patients with different grades: A preliminary study. *Indian J Cancer* **2015**, *52*, 580-585, doi:IndianJournalofCancer_2015_52_4_580_178395 [pii];10.4103/0019-509X.178395 [doi].
67. Lee, E.Q.; Muzikansky, A.; Drappatz, J.; Kesari, S.; Wong, E.T.; Fadul, C.E.; Reardon, D.A.; Norden, A.D.; Nayak, L.; Rinne, M.L., et al. A randomized, placebo-controlled pilot trial of armodafinil for fatigue in patients with gliomas undergoing radiotherapy. *Neuro Oncol* **2016**, *18*, 849-854, doi:now007 [pii];10.1093/neuonc/now007 [doi].
68. Suchorska, B.; Weller, M.; Tabatabai, G.; Senft, C.; Hau, P.; Sabel, M.C.; Herrlinger, U.; Ketter, R.; Schlegel, U.; Marosi, C., et al. Complete resection of contrast-enhancing tumor volume is associated with improved survival in recurrent glioblastoma-results from the DIRECTOR trial. *Neuro Oncol* **2016**, *18*, 549-556, doi:nov326 [pii];10.1093/neuonc/nov326 [doi].
69. Noll, K.R.; Bradshaw, M.E.; Weinberg, J.S.; Wefel, J.S. Relationships between neurocognitive functioning, mood, and quality of life in patients with temporal lobe glioma. *Psychooncology* **2017**, *26*, 617-624, doi:10.1002/pon.4046 [doi].
70. Odia, Y.; Iwamoto, F.M.; Moustakas, A.; Fraum, T.J.; Salgado, C.A.; Li, A.; Kreisl, T.N.; Sul, J.; Butman, J.A.; Fine, H.A. A phase II trial of enzastaurin (LY317615) in combination with bevacizumab in adults with recurrent malignant gliomas. *J Neurooncol* **2016**, *127*, 127-135, doi:10.1007/s11060-015-2020-x [doi];10.1007/s11060-015-2020-x [pii].
71. Lucchiari, C.; Botturi, A.; Manzini, L.; Masiero, M.; Pravettoni, G. Experiencing brain cancer: what physicians should know about patients. *Ecancermedicalscience* **2015**, *9*, 591, doi:10.3332/ecancer.2015.591 [doi];can-9-591 [pii].
72. Sagberg, L.M.; Solheim, O.; Jakola, A.S. Quality of survival the 1st year with glioblastoma: a longitudinal study of patient-reported quality of life. *J Neurosurg* **2016**, *124*, 989-997, doi:10.3171/2015.4.JNS15194 [doi].
73. Edelstein, K.; Coate, L.; Massey, C.; Jewitt, N.C.; Mason, W.P.; Devins, G.M. Illness intrusiveness and subjective well-being in patients with glioblastoma. *J Neurooncol* **2016**, *126*, 127-135, doi:10.1007/s11060-015-1943-6 [doi];10.1007/s11060-015-1943-6 [pii].
74. Armstrong, T.S.; Vera-Bolanos, E.; Acquaye, A.A.; Gilbert, M.R.; Ladha, H.; Mendoza, T. The symptom burden of primary brain tumors: evidence for a core set of tumor- and treatment-related symptoms. *Neuro Oncol* **2016**, *18*, 252-260, doi:nov166 [pii];10.1093/neuonc/nov166 [doi].
75. Okita, Y.; Narita, Y.; Miyahara, R.; Miyakita, Y.; Ohno, M.; Shibui, S. Health-related quality of life in long-term survivors with Grade II gliomas: the contribution of disease recurrence and Karnofsky Performance Status. *Jpn J Clin Oncol* **2015**, *45*, 906-913, doi:hyv115 [pii];10.1093/jjco/hyv115 [doi].
76. Jakola, A.S.; Sagberg, L.M.; Gulati, S.; Solheim, O. Perioperative quality of life in functionally dependent glioblastoma patients: A prospective study. *Br J Neurosurg* **2015**, *29*, 843-849, doi:10.3109/02688697.2015.1054355 [doi].
77. Oberg, J.A.; Dave, A.N.; Bruce, J.N.; Sands, S.A. Neurocognitive functioning and quality of life in patients with recurrent malignant gliomas treated on a phase Ib trial evaluating topotecan by convection-enhanced delivery. *Neurooncol Pract* **2014**, *1*, 94-100

II trial. *J Clin Oncol* **2011**, *29*, 3050-3055, doi:JCO.2011.34.8086 [pii];10.1200/JCO.2011.34.8086 [doi].
121. Jakola, A.S.; Unsgard, G.; Solheim, O. Quality of life in patients with intracranial gliomas: the impact of modern image-guided surgery. *J Neurosurg* **2011**, *114*, 1622-1630, doi:10.3171/2011.1.JNS101657 [doi].
122. Armstrong, T.S.; Vera-Bolanos, E.; Gning, I.; Acquaye, A.; Gilbert, M.R.; Cleeland, C.; Mendoza, T. The impact of symptom interference using the MD Anderson Symptom Inventory-Brain Tumor Module (MDASI-BT) on prediction of recurrence in primary brain tumor patients. *Cancer* **2011**, *117*, 3222-3228, doi:10.1002/cncr.25892 [doi].
123. Brada, M.; Stenning, S.; Gabe, R.; Thompson, L.C.; Levy, D.; Rampling, R.; Erridge, S.; Saran, F.; Gattamaneni, R.; Hopkins, K., et al. Temozolomide versus procarbazine, lomustine, and vincristine in recurrent high-grade glioma. *J Clin Oncol* **2010**, *28*, 4601-4608, doi:JCO.2009.27.1932 [pii];10.1200/JCO.2009.27.1932 [doi].
124. Ruge, M.I.; Ilmberger, J.; Tonn, J.C.; Kreth, F.W. Health-related quality of life and cognitive functioning in adult patients with supratentorial WHO grade II glioma: status prior to therapy. *J Neurooncol* **2011**, *103*, 129-136, doi:10.1007/s11060-010-0364-9 [doi].
125. Terasaki, M.; Eto, T.; Nakashima, S.; Okada, Y.; Ogo, E.; Sugita, Y.; Tokutomi, T.; Shigemori, M. A pilot study of hypofractionated radiation therapy with temozolomide for adults with glioblastoma multiforme. *J Neurooncol* **2011**, *102*, 247-253, doi:10.1007/s11060-010-0306-6 [doi].
126. Cheng, J.X.; Liu, B.L.; Zhang, X.; Lin, W.; Zhang, Y.Q.; Liu, W.P.; Zhang, J.N.; Lin, H.; Wang, R.; Yin, H. Health-related quality of life in glioma patients in China. *BMC Cancer* **2010**, *10*, 305, doi:1471-2407-10-305 [pii];10.1186/1471-2407-10-305 [doi].
127. Jones, L.W.; Mourtzakis, M.; Peters, K.B.; Friedman, A.H.; West, M.J.; Mabe, S.K.; Kraus, W.E.; Friedman, H.S.; Reardon, D.A. Changes in functional performance measures in adults undergoing chemoradiation for primary malignant glioma: a feasibility study. *Oncologist* **2010**, *15*, 636-647, doi:theoncologist.2009-0265 [pii];10.1634/theoncologist.2009-0265 [doi].
128. Kong, D.S.; Lee, J.I.; Kim, J.H.; Kim, S.T.; Kim, W.S.; Suh, Y.L.; Dong, S.M.; Nam, D.H. Phase II trial of low-dose continuous (metronomic) treatment of temozolomide for recurrent glioblastoma. *Neuro Oncol* **2010**, *12*, 289-296, doi:nop030 [pii];10.1093/neuonc/nop030 [doi].
129. Lucchiari, C.; Botturi, A.; Pravettoni, G. The impact of decision models on self-perceived quality of life: a study on brain cancer patients. *Ecancermedicalscience* **2010**, *4*, 187, doi:10.3332/ecancer.2010.187 [doi];can-4-187 [pii].
130. Jones, L.W.; Friedman, A.H.; West, M.J.; Mabe, S.K.; Fraser, J.; Kraus, W.E.; Friedman, H.S.; Tresch, M.I.; Major, N.; Reardon, D.A. Quantitative assessment of cardiorespiratory fitness, skeletal muscle function, and body composition in adults with primary malignant glioma. *Cancer* **2010**, *116*, 695-704, doi:10.1002/cncr.24808 [doi].
131. Mobed, K.; Liu, R.; Stewart, S.; Wrensch, M.; McCoy, L.; Rice, T.; Prados, M.; Chang, S. Quality of Life and Patterns of Use of Complementary and Alternative Medicines Among Glioma Patients. *J Support Oncol* **2009**, *7*, W23-W31.
132. Wang, M.; Cairncross, G.; Shaw, E.; Jenkins, R.; Scheithauer, B.; Brachman, D.; Buckner, J.; Fink, K.; Souhami, L.; Laperriere, N.J.I.J.o.R.O.B.P. Cognition and quality of life after chemotherapy plus radiotherapy (RT) vs. RT for pure and mixed anaplastic oligodendrogliomas: radiation therapy oncology group trial 9402. **2010**, *77*, 662-669.
133. Edvardsson, T.I.; Ahlstrom, G.I. Subjective quality of life in persons with low-grade glioma and their next of kin. *Int J Rehabil Res* **2009**, *32*, 64-70, doi:10.1097/MRR.0b013e32830bfa8c [doi];00004356-200903000-00008 [pii].
134. Corn, B.W.; Wang, M.; Fox, S.; Michalski, J.; Purdy, J.; Simpson, J.; Kresl, J.; Curran, W.J., Jr.; Diaz, A.; Mehta, M., et al. Health related quality of life and cognitive status in patients with glioblastoma multiforme receiving escalating doses of conformal three dimensional radiation on RTOG 98-03. *J Neurooncol* **2009**, *95*, 247-257, doi:10.1007/s11060-009-9923-3 [doi];10.1007/s11060-009-9923-3 [pii].
135. Gehring, K.; Sitskoorn, M.M.; Gundy, C.M.; Sikkes, S.A.; Klein, M.; Postma, T.J.; van den Bent, M.J.; Beute, G.N.; Enting, R.H.; Kappelle, A.C., et al. Cognitive rehabilitation in patients with gliomas: a randomized, controlled trial. *J Clin Oncol* **2009**, *27*, 3712-3722, doi:JCO.2008.20.5765 [pii];10.1200/JCO.2008.20.5765 [doi].
136. Kvale, E.A.; Murthy, R.; Taylor, R.; Lee, J.Y.; Nabors, L.B. Distress and quality of life in primary high-grade brain tumor patients. *Support Care Cancer* **2009**, *17*, 793-799, doi:10.1007/s00520-008-0551-9 [doi].
137. Jones, L.W.; Cohen, R.R.; Mabe, S.K.; West, M.J.; Desjardins, A.; Vredenburgh, J.J.; Friedman, A.H.; Reardon, D.A.; Waner, E.; Friedman, H.S. Assessment of physical functioning in
